# Successful video-assisted thoracoscopic surgery in prone position in patients with esophageal cancer and aberrant right subclavian artery: report of three cases

**DOI:** 10.1186/s40792-017-0360-9

**Published:** 2017-07-28

**Authors:** Koji Shindo, Eishi Nagai, Toshinaga Nabae, Toru Eguchi, Taiki Moriyama, Kenoki Ohuchida, Tatsuya Manabe, Takao Ohtsuka, Yoshinao Oda, Makoto Hashizume, Masafumi Nakamura

**Affiliations:** 1 Departments of Surgery and Oncology, Graduate School of Medical Sciences, Kyushu University, 3-1-1 Maidashi, Higashi-ku, Fukuoka City, Fukuoka Japan; 2 Center for Advanced Medical Innovation, Graduate School of Medical Sciences, Kyushu University, 3-1-1 Maidashi, Higashi-ku, Fukuoka City, Fukuoka 812-8582 Japan; 3 Department of Surgery, Japan Community Health care Organization (JCHO) Kyushu Hospital, 1-8-1 Kishinoura, Yahatanishi-ku, Kitakyushu City, Fukuoka 806-8501 Japan; 4 Department of Surgery, Harasanshin Hospital, 1-8 Daihakucho, Hakata-ku, Fukuoka City, Fukuoka 812-0033 Japan; 5 Department of Anatomic Pathology, Graduate School of Medical Sciences, Kyushu University, 3-1-1 Maidashi, Higashi-ku, Fukuoka City, Fukuoka 812-8582 Japan

**Keywords:** Video-assisted thoracoscopic surgery, Prone position, Aberrant right subclavian artery, Nonrecurrent right inferior laryngeal nerve, Esophageal cancer

## Abstract

**Background:**

An aberrant right subclavian artery (ARSA) with an associated nonrecurrent right inferior laryngeal nerve (NRILN) is a relatively rare anomaly that occurs at a frequency of 0.3 to 2.0% of the general population. NRILN has been mainly documented in the head and neck region; it has been rarely described in patients with esophageal cancer, especially those undergoing thoracoscopic surgery. Video-assisted thoracoscopic surgery for esophageal cancer (VATS-E) is becoming more widespread as a reliable minimally invasive surgical procedure associated with reduced perioperative complications.

**Case presentation:**

Herein, we report three cases of esophageal cancer with ARSA and NRILN which underwent successful VATS-E. Case 1, a 53-year-old male who had early stage esophageal cancer was performed VATS-E. Upper gastrointestinal (GI) series showed “Bayonet sign” (T1aN0M0, pStageIA in UICC). Case 2, a 75-year-old male who had advanced esophageal cancer was performed neoadjuvant chemotherapy and following VATS-E. This case had right thoracic duct and “Bayonet sign” on upper GI series (T1bN2M0, pStage IIIA in UICC). Case3, a 72-year-old male who had advanced esophageal cancer was performed neoadjuvant chemotherapy and following VATS-E (T3N2M0, pStageIIIB in UICC). All of these three cases were performed VATS-E and discharged without any complication.

**Conclusion:**

VATS-E in the prone position is a feasible procedure that can reduce the risk of complications with an enlarged and clear view, and knowledge of this type of anomaly is very important for surgeons who perform esophagectomy.

## Background

Video-assisted thoracoscopic surgery for esophageal cancer (VATS-E) is a reliable minimally invasive surgery that provides an enlarged view and reduced perioperative complications such as wound infection and pulmonary complications [1]. All involved staff members can share the enlarged, detailed view, which is useful for quality control of surgical procedures. Moreover, use of the prone position during VATS-E helps to keep the operating field clear by avoiding the pooling of blood and leachate. However, lymphadenectomy in the upper mediastinum is still a stressful procedure for many surgeons because of the difficulty of exploration for the recurrent nerve. Injury to this nerve may decrease the patient’s quality of life because of the development of hoarseness and an increased risk of aspiration pneumonia. A nonrecurrent right inferior laryngeal nerve (NRILN) is a relatively rare anomaly of the recurrent nerve that is associated with an aberrant right subclavian artery (ARSA). It is essential that surgeons who perform esophagectomy are familiar with the anatomy of the vessels and nerves in this region, including their variations. We herein describe three patients with esophageal cancer and an NRILN with an associated ARSA who underwent VATS-E and highlight the key points to remember in such cases.

## Case Presentation

### Case 1

**Fig. 1:**
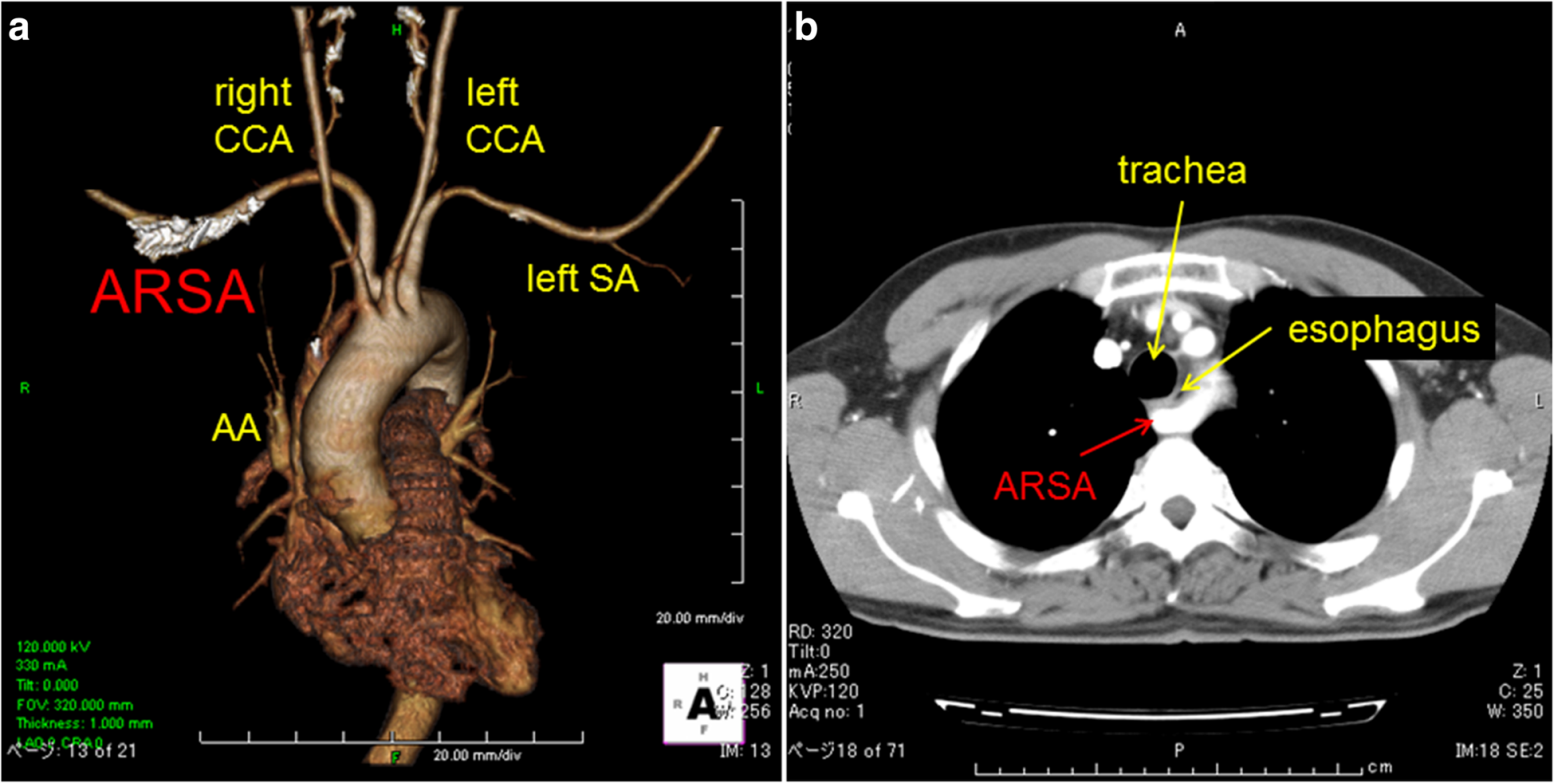
Case 1. **a** Three-dimensional and **b** enhanced computed tomography scan showed an aberrant right subclavian artery arising from the dorsal side of the distal descending aorta and passing through the retroesophageal space. *AA* aortic arch, *SA* subclavian artery, *CCA* common carotid artery

**Fig. 2:**
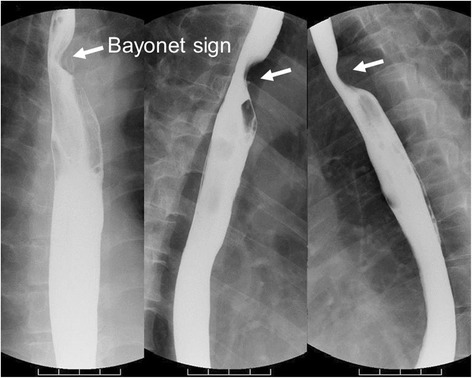
Case 1. Upper gastrointestinal series showed the bayonet sign, which indicated compression of the aberrant right subclavian artery (*arrow*)

A 53-year-old man presented to the hospital with general fatigue and epigastralgia. Upper gastrointestinal (GI) endoscopy showed a hemorrhagic duodenal ulcer, which was medically treated. Secondary upper GI endoscopy a few weeks later revealed healing of the ulcer and early-stage cancer in the middle thoracic esophagus. The cancerous lesion was 8 cm in length. A biopsy specimen revealed squamous cell carcinoma. Submucosal invasion was identified by endoscopic ultrasound, and the patient was then referred to the surgical department. Enhanced computed tomography (CT) demonstrated an ARSA originating from the aortic arch, passing behind the esophagus, and ascending on the right side of the esophagus (Fig. 1). An upper GI series showed the bayonet sign, which indicated compression of the esophagus by the ARSA (Fig. 2). However, the cancerous lesion was not found. The patient underwent VATS-E with no complications. The normal right recurrent nerve was not identified in the upper mediastinum, but an NRILN was found in the neck and preserved without injury. Gastric tube reconstruction was performed through the retrothoracic route. After pathologic examination, the esophageal cancer was diagnosed as squamous cell carcinoma (Mt, Type 0-IIb + IIc, T1a (LPM), N0, M0: pStage 0). According to the TNM classification seventh edition (Union for International Cancer Control), this tumor was T1aN0M0, pStageIA.

### Case 2

**Fig. 3:**
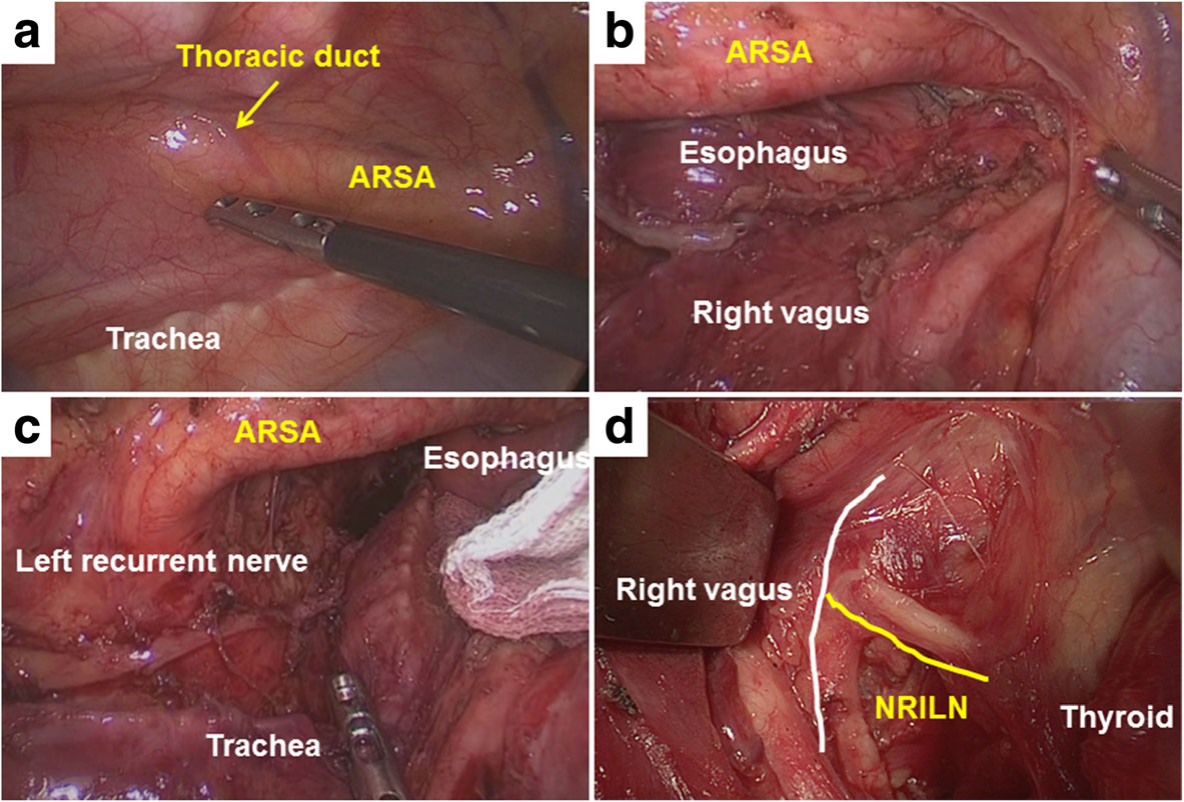
Case 2, intraoperative findings. **a** The right thoracic duct coursed across the aberrant right subclavian artery and esophagus, running into the right venous angle. **b** The right recurrent nerve was not evident. **c** The left recurrent nerve was normal, and the space in the upper mediastinum on left side was restricted by the aberrant right subclavian artery. **d** In the neck, the nonrecurrent right inferior laryngeal nerve headed into the larynx directly from the vagus trunk

A 75-year-old man presented to the hospital with dysphagia and precordial pain. Upper GI endoscopy showed advanced-stage esophageal cancer at the upper to middle thoracic esophagus. A biopsy specimen revealed squamous cell carcinoma. Enhanced CT demonstrated advanced esophageal cancer with multiple enlarged lymph nodes and the presence of an ARSA. On an upper GI series, advanced esophageal cancer was identified with the bayonet sign. Neoadjuvant chemotherapy (NAC) using fluorouracil + cisplatin was performed twice. A CT scan after the NAC showed that the tumor and enlarged lymph nodes had improved. The patient underwent VATS-E with no complications. The normal right recurrent nerve was not evident (Fig. 3a–c), but an NRILN was detected in the neck and preserved without injury (Fig. 3d). The thoracic duct was located on the right side and ran across the esophagus and into the right venous angle (Fig. 3a). Gastric tube reconstruction was performed through the retrothoracic route. After pathologic examination, the esophageal cancer was diagnosed as squamous cell carcinoma (MtUt, Type 0-IIa, T1b(SM), N3, M0: ypStage III), or T1bN2M0, pStage IIIA in UICC.

### Case 3

A 72-year-old man presented to the hospital with dysphasia. Upper GI endoscopy and an upper GI series showed advanced-stage esophageal cancer in the middle thoracic esophagus. The bayonet sign was not evident. A biopsy specimen revealed squamous cell carcinoma. Enhanced CT showed an ARSA passing behind the esophagus. NAC using fluorouracil + cisplatin was performed twice, and VATS-E was then performed with no complications. The thoracic duct was located behind the ARSA in the upper mediastinum. The normal right recurrent nerve was absent, and an NRILN was originating directly from the right vagus nerve at the neck. After pathologic examination, the esophageal cancer was diagnosed as squamous cell carcinoma (Mt, Type 5, T3 (AD), N2, M0: ypStage III) or T3N2M0, pStageIIIB in UICC.

These three cases are summarized in Table 1. All patients underwent VATS-E with no complications or transfusions.

**Table 1:** Summary of our three patients with esophageal cancer and an aberrant right subclavian artery with an associated nonrecurrent right inferior laryngeal nerve

Case	Gender	Age	Tumor location	NAC	Final stage	Modality for preoperative detection of ARSA	Bayonet sign	Reconstruction route of gastric tube	Thoracic duct	Classification of NRILN (Toniato)	Operative time (min)	Estimated Blood loss (g)	Complication
1	Male	53	Mt	None	Stage IA	CT	Yes	Retrothoracic	Normal	Type 2A	450	60	None
2	Male	75	MtUt	5-FU with cisplatin	Stage IIIA	CT	Yes	Retrothoracic	Right side	Type 1	722	100	None
3	Male	72	Mt	5-FU with cisplatin	Stage IIIB	CT	No	Retrothoracic	normal	Type 2A	582	175	None

*NAC* neoadjuvant chemotherapy

### Discussion

Esophagectomy with three-field lymph node dissection remains the standard treatment for esophageal cancer even in the relatively early stage, although this surgery must sometimes be preceded by chemoradiation therapy. Radical lymph node dissection is essential because it affects patient outcomes [2–5], especially in patients with upper and middle thoracic esophageal cancer [3, 6, 7]. However, vocal cord paralysis due to injury of the recurrent nerve may cause aspiration pneumonia, which is a severe complication after esophagectomy with lymphadenectomy [3]. Therefore, surgeons must accurately identify and preserve the recurrent nerve.

Some reports described that VATS-E is safe and better than an open esophagectomy in terms of blood loss, postoperative recovery, less respiratory complications, and also improved overall survival condition [8, 9]. Moreover, as for the position during thoracoscopy, pooled analysis suggests that VATS-E in the prone position is superior to lateral decubitus position with reduced pulmonary complications, estimated blood loss, and increased mediastinal lymph node harvest [10]. Palanivelu et al. [11] reported that VATS-E in the prone position provides a clear operative field using gravity and carbon dioxide pneumothorax. VATS-E in the prone position was recently reported to exhibit usability and feasibility for successful esophagectomy and lymphadenectomy [11, 12].

The enlarged view makes small vessels clearly visible, and intraoperative pooling of blood and leachate does not disturb the view because the operative field is not at the bottom in this prone position (unlike in the left lateral position). Gentle rotation of the trachea using a rumpled piece of gauze on the membranous portion of the trachea also helps to obtain a clear and safe view. As shown in Table 1, we successfully performed VATS-E in the prone position without transfusion or intraoperative morbidity. The mean blood loss was only 112 g (range, 60–175 g), which favorably compares with the data in other reports of VATS-E [1, 8] despite the existence of an ARSA and the performance of three-field lymphadenectomy.

Generally, the right fourth branchial arch becomes the right subclavian artery, and the left sixth branchial arch changes into the arterial duct. The laryngeal nerves in the neck are pulled into the thoracic cavity as the heart descends. These nerves then exist as recurrent nerves that turn around the right subclavian artery and aortic arch, respectively. On the right side, abnormal involution of the fourth branchial arch occasionally occurs in the early embryonic stage. In this situation, the ARSA forms by a compensatory seventh intersegmental artery. Basically, the ARSA arises from the dorsal side of the distal descending aorta and passes through the retroesophageal space. Epstein and Debord [13] discussed the course of the ARSA, which may pass between the trachea and esophagus in extremely rare cases. As a result, the right laryngeal nerve directly runs into the larynx from the vagus trunk because it was not pulled into the thoracic cavity [14, 15].

The presence of an ARSA is a relatively rare anomaly, occurring in about 0.3 to 2.0% of the general population [13, 16–18]. It is associated with an NRILN [14], as mentioned above. CT can be used to clearly detect an ARSA, which indicates the likely presence of an NRILN [14]. Most patients with an ARSA remain asymptomatic; however, 10% of adult patients with an ARSA have compressive symptoms [19] such as dysphagia lusoria with a bayonet sign on upper GI series [20]. Moreover, 60% of patients with an ARSA also have a Kommerell diverticulum [21], which also can cause compressive symptoms [21–23]. NRILN has mainly been documented in the head and neck region; however, it has also been rarely reported in patients with esophageal cancer, especially those undergoing thoracoscopic surgery [6, 24]. Patients with an NRILN have a much higher possibility of iatrogenic vocal cord paralysis due to intraoperative injury of the recurrent nerve [18]. Thus, surgeons who perform esophagectomy must pay close attention to patients with an ARSA and associated NRILN and the related complications that may occur.

If a normal recurrent nerve is not identified intraoperatively, the surgeon may have difficulty in performing lymphadenectomy around the vagus nerve. Knowledge of such an embryologic variation plays an essential role in minimizing intraoperative injury during lymphadenectomy for esophageal cancer [25–28]. However, surgeons must keep the possibility of coexistence with a normal recurrent nerve in mind because some authors have described patients with a coexisting right recurrent inferior laryngeal nerve and NRILN [26, 27]. Some patients also had an associated ARSA [26]. Intraoperative nerve monitoring might be helpful in such cases [29].

Various problems may occur during esophagectomy in patients with an ARSA and NRILN. First, the working space may be restricted by the protruding ARSA in the left upper mediastinum, making completion of lymphadenectomy difficult. Second, the thoracic duct runs behind the esophagus and across the ARSA and esophagus to the right side at a relatively high rate [30] because the ARSA physically prevents the normal course of the thoracic duct and forces it to deviate toward the right side. Finally, as reported by Toniato et al. [18], the NRILN exhibits three patterns as it heads into the larynx: in type 1, it runs together with the vessels of the superior thyroid peduncle; in type 2A, it follows a transverse path parallel to the inferior thyroid artery; and in type 2B, it runs under the trunk or between the branches. Surgeons must carefully consider the course of the NRILN to avoid misconception. VATS-E in the prone position can help to confirm and preserve the nerves in the thoracic space because of the shared and enlarged view, especially in patients with anatomical variations.

## Conclusions

In conclusion, we have herein reported three cases of successful VATS-E for treatment of esophageal cancer in patients with an ARSA and NRILN with no morbidity and discussed key points to be noted when performing esophagectomy. Knowledge of this type of anomaly is very important in esophagectomy. VATS-E in the prone position can help to reduce the risk of complications with an enlarged and clear view.

## Abbreviations

ARSA: An aberrant right subclavian artery
CT: Computed tomography
GI: Gastrointestinal
NAC: Neoadjuvant chemotherapy
NRILN: Nonrecurrent right inferior laryngeal nerve
UICC: Union for International Cancer Control
VATS-E: Video-assisted thoracoscopic surgery for esophageal cancer
